# Rab27a dependent exosome releasing participated in albumin handling as a coordinated approach to lysosome in kidney disease

**DOI:** 10.1038/s41419-020-2709-4

**Published:** 2020-07-08

**Authors:** Ye Feng, Xin Zhong, Tao-Tao Tang, Cui Wang, Li-Ting Wang, Zuo-Lin Li, Hai-Feng Ni, Bin Wang, Min Wu, Dan Liu, Hong Liu, Ri-Ning Tang, Bi-Cheng Liu, Lin-Li Lv

**Affiliations:** Institute of Nephrology, Zhongda Hospital, Southeast University School of Medicine, Nanjing, Jiangsu Province 210009 China

**Keywords:** End-stage renal disease, Toxin-induced nephropathy

## Abstract

Exosomes are increasingly recognized as vehicles of intercellular communication. However, the role of exosome in maintaining cellular homeostasis under stress conditions remained unclear. Here we show that Rab27a expression was upregulated exclusively in tubular epithelial cells (TECs) during proteinuria nephropathy established by adriamycin (ADR) injection and 5/6 nephrectomy as well as in chronic kidney disease patients, leading to the increased secretion of exosomes carrying albumin. The active exosome production promoted tubule injury and inflammation in neighboring and the producing cells. Interferon regulatory factor 1 (IRF-1) was found as the transcription factor contributed to the upregulation of Rab27a. Albumin could be detected in exosome fraction and co-localized with exosome marker CD63 indicating the secretion of albumin into extracellular space by exosomes. Interestingly, inhibition of exosome release accelerated albumin degradation which reversed tubule injury with albumin overload, while lysosome suppression augmented exosome secretion and tubule inflammation. Our findings revealed that IRF-1/Rab27a mediated exosome secretion constituted a coordinated approach to lysosome degradation for albumin handling, which lead to the augment of albumin toxicity as a maladaptive response to maintain cell homeostasis. The findings may suggest a novel therapeutic strategy for proteinuric kidney disease by targeting exosome secretion.

## Introduction

Exosomes are nanosized vesicles released by fusion of an intermediate endocytic compartment, the multivesicular body, with the plasma membrane^1^. During the past decades, great efforts have been made to elucidate the role of exosomes as vehicles for intercellular communication^2,3^. However, as the intracellular interconnected vesicular network, the cross talk between exosome secretion and cellular lysosome degradation to maintain cellular homeostasis remained unclear^4^.

Albuminuria, is not only a common manifestation of kidney injury, but also the independent mediator in accelerating kidney disease progression via aggravating tubular epithelial cell (TEC) injury and tubulointerstitial inflammation^5–7^. Although the importance of urinary albumin in disease progression is recognized, the adaptive response of TECs in the presence of excessive albumin remains to be determined. TECs are well adapted as reclaimer of large molecules, such as albumin, that filtered by glomeruli^8^. In normal conditions, the filtered albumin is efficiently reabsorbed by TECs via megalin, cubilin or amnionless (AMN) receptor, and is subsequently degraded into amino acids within lysosomes to maintain cellular homeostasis^9^. Alternatively, Fc receptor might mediate the reabsorption of albumin and protect albumin from lysosomal degradation by transcytosis^10,11^. However, in condition of albumin overload, excessive reabsorption of albumin into TECs with insufficient lysosomal degradation may lead to severe tubular toxicity and inflammatory response^12^, while the underlying mechanism remains to be clarified.

Under stress conditions, exosomes can be secreted as paracrine or autocrine signals to modulate the activity and fate of recipient cells as well as the producing cells^1^. Previous studies showed that renal TECs released increasing number of exosomes in the setting of hypoxia and albuminuria, which transferred to target cells with functional cargoes and promote renal inflammation and fibrosis^13–15^. However, the regulatory mechanisms underlying the active exocytosis of exosomes remain largely unknown. Importantly, whether the increasing release of exosomes represents a response to albumin overload to maintain cellular hemostasis warrants further investigation.

Accumulating evidence suggests that exosomes may serve as a quality control mechanism in maintaining cellular homeostasis by selective release of intracellular harmful components during stress or pathological conditions^4,16^. Misfolded soluble proteins could be encapsulated and released in exosomes^17^. Moreover, recent study revealed that exosome release serves as an alternative disposal pathway to the lysosome^18^. Thus, it would be interesting to observe the role of exosomes in albumin handling for TECs, which might provide important clues to understand the mechanism of albumin induced tubular injury and tubulointerstitial inflammation.

In our study, we identified that IRF1/Rab27a mediated the increasing secretion of exosomes upon albumin exposure. Exosome release represented an alternative response in albumin handling through excretion of excessive albumin when lysosome degradation is insufficient, and the two endo-lysosomal pathways are cross regulated. Importantly, our results revealed that exocytosis of albumin via exosomes lead to a maladaptive response that augmented albumin toxicity to tubules and promoted tubulointerstitial inflammation.

## Materials and methods

### Animal models

Six to eight weeks male C57BL/6J mice and Sprague Dawley (SD) rats were purchased from Beijing Vital River Laboratory Animal Technology Co. Ltd. The murine experiments were approved by the Ethics Committee of Southeast University. Sample size estimation was performed before experiments. Proteinuric kidney injury model was induced by adriamycin (ADR) administration as described previously^19^. Briefly, C57BL/6J mice (*n* = 6 per group) were randomly injected intravenously with 18 mg/kg body weight ADR dissolved in ddH_2_O or equal volume of ddH_2_O. Urine was collected weekly, and mice were sacrificed on day 23 after ADR treatment. To specifically knockdown Rab27a in kidney, 2 × 10^7^ TU of lentivirus Rab27a shRNA (5′-GATGCACGCGTACTGTGAA-3′, constructed by Genechem, Shanghai) or negative control (5′-TTCTCCGAACGTGTCACGT-3′) was intrarenal injected into the renal cortex of C57BL/6J mice. Renal cortex was harvested from all groups of mice. The chronic kidney disease model was established in rats by performing surgical 5/6 subtotal nephrectomy as reported before^20^. Male rats (*n* = 5 per group) were assigned to sham-operated or nephrectomy groups. The animals were subjected to right nephrectomy and resection of the upper and lower one-third of the left kidney. The rats were sacrificed at week 16 and serum samples, 24-h urine, and kidney tissues were collected.

### Cells

mTECs (immortalized mouse tubular epithelial cells) were gift from Prof. Hui-Yao Lan, The Chinese University of Hong Kong. mTECs were cultured in DMEM/F12 (Hyclone, USA) supplemented with 10% fetal bovine serum (Gibco, USA), penicillin and streptomycin (Gibco, USA).

### CKD Patients and controls

The study was approved by the Ethics Committee of Zhongda Hospital, Southeast University. Normal kidney tissues from patients with renal carcinoma were used as controls (*n* = 4). CKD patients including IgA nephropathy, membranous nephropathy, diabetic nephropathy, and lupus nephritis were enrolled (*n* = 8). All the laboratory measurements, including blood urea nitrogen (BUN), serum creatinine (SCr), uric acid (UA), 24-h proteinuria, were obtained on the day of urine sample collection. Informed consent was obtained from all subjects.

### Renal tubule isolation

Renal tubules were isolated from kidneys of mice by differential sieving, as described previously^21^. Briefly, male C57BL/6J mice were anesthetized and the kidneys were removed and placed in cold sterile DMEM solution. After removing the renal capsules and medulla, the cortical layers were dissected and minced into pieces. The kidney tissues were ground with an 80-mesh stainless steel sieve, followed by filtering through a 100-mesh steel sieve. Renal tubule fractions in 100-mesh steel sieve were collected and centrifuged at 1500 rpm for 5 min. The supernatant was eliminated, and the sediment washed once with sterile DMEM was collected for further protein and mRNA expression detection.

### Exosome purification

TECs were treated with BSA in DMEM/F12 medium without serum for 24 h, the cells were then washed with sterile PBS for 3 times, and cultured in serum and BSA free medium allowing for secretion of exosomes. After another 24 h, supernatants were harvested and subjected to differential centrifugation. To isolate exosomes from renal tubules, 0.1 g fresh mouse renal tubules were dissected and incubated in DMEM solution that contained trypsin and collagenase for 2 h at 37 °C with gentle rotation. The reaction was stopped by exosome-free FBS. The medium was harvested for exosome purification as previously reported^20^. Briefly, the collected medium was centrifuged at 2000 × *g* for 20 min and 13,500 × *g* for 20 min to remove cellular debris and large extracellular vesicles, respectively. Supernatants were further centrifuged for 2 h with a Type 70 Ti rotor (Beckman Optimal-100 XP) at 200,000 × *g* at 4 °C. Pellets were resuspended with sterile PBS, and were centrifuged again by at 200,000 × *g*. The washed pellets were reconstituted in 200 μl PBS and stored at −80 °C for further analysis.

### OptiPrep^TM^ density gradient centrifugation

Purification of exosomes by density gradient was described as previously^22^. OptiPrep^TM^ aqueous iodixanol solution (60% w/v) was mixed with a homogenization buffer (containing 0.25 M sucrose, 10 mM Tris-HCl, 1 mM EDTA, pH7.4) to prepare the solutions of 5, 10, 20, and 40% iodixanol. The gradient was set up by layering 3 ml of 40, 20, and 10% iodixanol solutions and 2.8 ml of 5% solution in a 13.2 ml ultra-clear tube (Beckman Coulter). Exosome pellets obtained by ultracentrifugation from TECs were resuspended and overlaid onto the top of the gradient, with centrifugation for 18 h at 100,000 × *g* and 4 °C (SW41Ti rotor, Beckman Coulter). Individual fractions of 1 ml were collected from the top of the gradient and diluted with 20 ml in PBS and centrifuged for 2 h at 200,000 × *g* and 4 °C. The pellets were resuspended in 100 µl PBS and stored at −80 °C for further analysis. A standard curve was made by the absorbance values at 340 nm of 5, 10, 20, and 40% iodixanol solutions to estimate the density of each fraction collected from samples.

### Exosome characterization

The exosome pellets isolated from renal tissue or culture medium were applied to 200-mesh nickel grids, stained with 2% phosphotungstic acid for 5 min and air-dried. Exosomes were detected using a transmission electron microscope (HT 7700; Hitachi High- Tech, Tokyo, Japan) at 80 kV. Exosome number were quantified using the EXOCET Quantification Assay kit (System Biosciences, Mountain View, CA) according to the manufacturer’s protocols, by measuring the activity of exosome acetylcholinesterase.

### Confocal analysis of DQ-albumin degradation in vitro and in vivo

A dye quenched (DQ)-albumin (D-12051, Molecular Probes, USA) was used for the detection of the degradation capacity of lysosomes. DQ-albumin is labeled with self-quenched fluorescent dye that emits fluorescence upon being digested by lysosomal proteases^23^. TECs were seeded on confocal dish, grown to confluency and serum deprived. After treated with BSA or PBS for 24 h, TECs were exposed to DQ-BSA for 4 h. Cells were fixed and then analyzed by confocal microscopy (Olympus, FV1000).

### Immunofluorescence staining

TECs were harvested with 4% paraformaldehyde fixation and then permeabilized with 0.25% Triton X-100 for 5 min followed by incubation of antibodies. For immunofluorescence staining of kidney tissue, the kidney sections were incubated with primary antibodies to Rab27a (ab55667, Abcam, UK), albumin (16475-1-AP, Proteintech, USA), CD63 (ab213090, Abcam, UK), MCP-1 (ab9899, Abcam, UK), IRF-1 (sc514544, Santa Cruz, USA) overnight at 4 °C, followed by incubation with anti-mouse (ab150116. Abcam, UK) or anti-rabbit (bs-0295D, Bioss, China) secondary antibody for 1 h. All samples were treated with DAPI dye for nuclear staining.

### Rab27a knockdown and overexpression in vitro

Transfection was performed until TECs were cultured to 80–90% confluence, according to the manufacture’s protocol (GenePharma, China). Briefly, the Rab27a siRNA oligonucleotide (sense 5′-GAUGCACGCGUACUGUGAATT-3′, antisense 5′-UUCACAGUACGCGUGCAUCTT-3′), KIBRA siRNA oligonucleotide (sense 5′- GCACAGAGACCAGGUACUUTT, antisense 5′-AAGUACCUGGUCUCUGUGCTT) or NC (Sense 5′-UUCUCCGAACGUGUCACGUdTdT-3′, antisense 5′-ACGUGACACGUUCGGAGAAdTdT-3′) or recombinant plasmids for Rab27a overexpression or scramble (commercially constructed by Genechem Co., Ltd, Shanghai, China) and Lipofectamine 2000 (Invitrogen, USA) were mixed with Opti-MEM (Gibco, USA) respectively at room temperature for 5 min and applied to cells with serum-free culture medium.

### Western blotting

For western blotting analysis, samples were lysed in Lysis Buffer (Thermo, USA) with 1% protease inhibitor. Protein concentration was analyzed using a BCA protein assay kit (Thermo) according to the manufacturer’s protocol. Samples were loaded and separated by 10% SDS-PAGE, followed by transferring onto PVDF membranes (Millipore, USA). After blocking with 5% milk in TBS-T for 1 h, membranes were probed with primary antibodies as follows: Alix (sc53540, Santa Cruz, USA; 1:500), CD63 (ab213090, Abcam, UK; 1:2000), CD81 (10037, Cell Signaling Technology, USA; 1:2000), CD9 (ab92726,Abcam, UK; 1:1000), Rab27a (ab55667, Abcam, UK; 1:2000), IRF-1 (sc514544, Santa Cruz, USA; 1:2000), β-actin (sc47778, Santa Cruz, USA; 1:2000), BSA (sc32816, Santa Cruz, USA; 1:200), cathepsin B (ab58802, Abcam, UK; 1:1000) and cathepsin D (ab6313, Abcam, UK; 1:1000). The membranes were then incubated with secondary horseradish peroxidase-conjugated antibodies (Cell Signaling Technology) and visualized with ECL Advance system (GE Healthcare, UK). Intensity values expressed as the relative protein expression were normalized to β-actin.

### Quantitative real-time PCR

The total RNA was extracted from renal cortex of mice and cultured cells using RNAiso Plus (Takara, Japan), according to the manufacturer’s instructions. Reverse transcription and quantitative renal-time PCR were performed using a PrimeScript RT reagent kit and SYBR Premix Ex Taq (Takara, Japan). All the data are reported as mean ± standard error of mean (S.E.M) normalized to GAPDH. The primer sequences were listed in Table 1.

**Table 1:** Primers for quantitative RT-PCR.

Primer	Forward	Reverse
Rab27a	TTCCTGCTTCTGTTCGACCT	GCTTATGTTTGTCCCGTTGG
IRF-1	GCTGGTCTTGCTGGGTACTG	CCGTCCTGTCCTTCAGTCAT
Hrs	ACCTTCGAGCGTCTCCTAGA	GGCCACAGTTCTTTACCACA
nSMase2A	AGTACGAGGACCGGGTTTCT	AGTGCTTCTTTGGCTGGTTC
MCP-1	CTTCTGGGCCTGCTGTTCA	CCAGCCTACTCATTGGGATCA
IL-6	AAAGAGTTGTGCAATGGCAATTCT	AAGTGCATCATCGTTGTTCATACA
TNF-α	CATCTTCTCAAAATTCGAGTGACAA	TGGGAGTAGACAAGGTACAACCC
KIM-1	TCAGAAGAGCAGTCGGTACAAC	TGTAGCTGTGGGCCTTGTAGT
LCN2	GCCCTGAGTGTCATGTGTCT	GAACTGATCGCTCCGGAAGT
GAPDH	GCATGGCCTTCCGTGTTC	GATGTCATCATACTTGGCAGGTTT

### Statistical analysis

Data are expressed as the mean ± standard error of mean (S.E.M) of each group. Statistical significance was determined using a Student’s *t*-test and one-way analysis of variance in SPSS 20.0 statistical software. A two-sided *p* value less than 0.05 were considered statistically significant.

## Results

### Tubular exosome secretion is increased carrying albumin in proteinuric kidney disease model induced by ADR injection and 5/6 nephrectomy

**Fig. 1:**
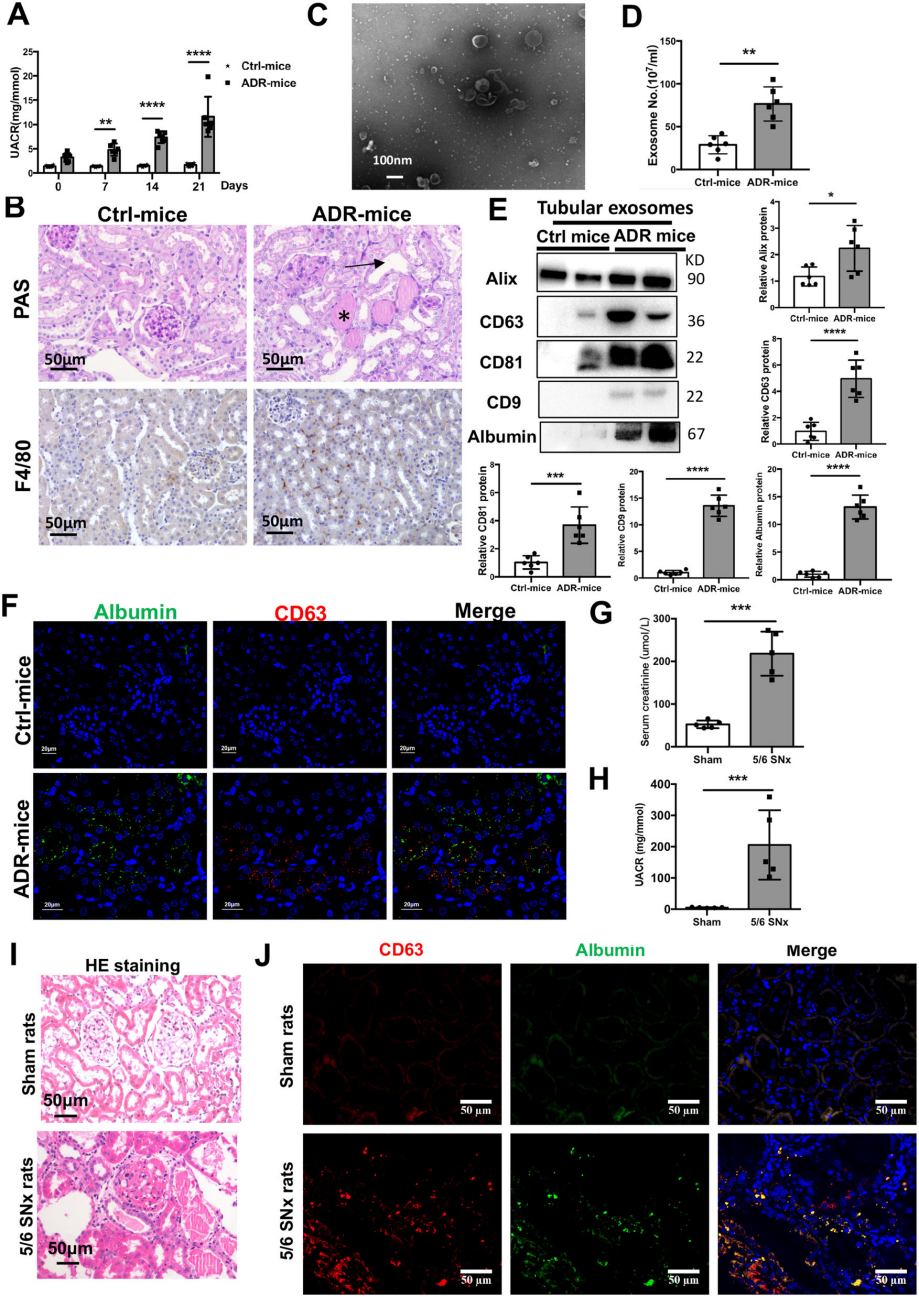
Tubular exosome secretion carrying albumin is increased in proteinuric kidney disease model. **a** Urine protein measured at 0, 7, 14, and 21 days after injection with ADR or ddH_2_O. Proteinuria presented at 7, 14, and 21 days. **b** Periodic acid-Schiff staining and immunohistochemistry analysis of F4/80 of the mice kidney. Representative images of renal tissues from mice injected with ddH_2_O or ADR are shown. Significant tubular injury and protein cast were indicated with arrows and stars in ADR-induced mice. Increased F4/80 positive macrophages infiltrated in diseased kidney. Scale bars:50 μm. **c** Representative electron microscopy image of tubular exosomes isolated from mice with ADR injection. Scale bars:100 nm. **d** Quantification of tubular exosomes from ADR or control mice using EXOCET assay kit. **e** Representative western blotting and quantification of exosome markers (Alix, CD63 and CD81, CD9) and albumin. The bar graph shows protein expression from ADR-induced mice compared with levels in control mice (represented by 1-fold). **f** Representative images of immunofluorescence for albumin (green) and CD63 (red) in kidney. Scale bar: 20 μm. **p < 0.01, ***p < 0.001, ****p < 0.0001 versus control group. Ctrl, control; ADR, adriamycin. *n* = 6 for each group of mice. **g**, **h** Serum levels of creatinine and urinary albumin-to-creatinine ratios in rats with sham or 5/6 nephrectomy at 16 weeks after surgery. **i** HE staining of the rat kidney. Representative images of renal tissues from rats with sham or 5/6 nephrectomy are shown. Scale bars: 50 μm. **j** Representative images of immunofluorescence for albumin (green) and CD63 (red) in kidney. Scale bar: 50 μm. ***p < 0.001 vs sham rats. SNx, subtotal nephrectomy. *n* = 5 for each group of rats.

To characterize exosome secretion in proteinuric kidney injury, firstly, we established ADR-induced proteinuric kidney disease model as previously reported^24^. Mice developed significant proteinuria 7 days after ADR injection (Fig. 1a). Histologically, tubular collapse, intratubular protein casts and F4/80 positive macrophages infiltration was found at 3 weeks (Fig. 1b). Our previous studies showed that exosomes production was induced significantly in ADR-kidney^15^. To further investigate the secretion of exosomes from tubules, exosomes were purified from isolated tubular fractions. As expected, the size and shape of the purified vesicles showed typical morphology of exosomes (Fig. 1c). Interestingly, tubules from ADR-injected mice displayed more active exosome secretion than Ctrl mice, shown by EXOCET assay and western blot analysis of exosomes markers (Fig. 1d, e), indicating that exosome release was enhanced in renal tubules under proteinuria conditions. Notably, albumin was detected in tubular exosomes secreted from ADR-injected mice (Fig. 1e), which was also co-localized with CD63-labled multivesicular bodies (MVB) in diseased kidney, especially in area of tubule (Fig. 1f).

To explore the role of exosomes in albumin handling in another proteinuric kidney injury model, rats with 5/6 nephrectomy were used. Sixteen weeks after surgery, serum creatinine and proteinuria (Fig. 1g, h) were markedly increased in 5/6 nephrectomy rats, accompanying with histological tubular injury and protein cast shown by HE staining (Fig. 1i). Interestingly, co-localization of CD63 and albumin were observed in tubules of 5/6 nephrectomy rats (Fig. 1j). Thus, the data collectively suggested that exosome secretion was increased carrying albumin in tubule during proteinuric kidney injury.

### Rab27a expression as well as inflammation response were induced in injured tubules in proteinuric kidney injury model

**Fig. 2:**
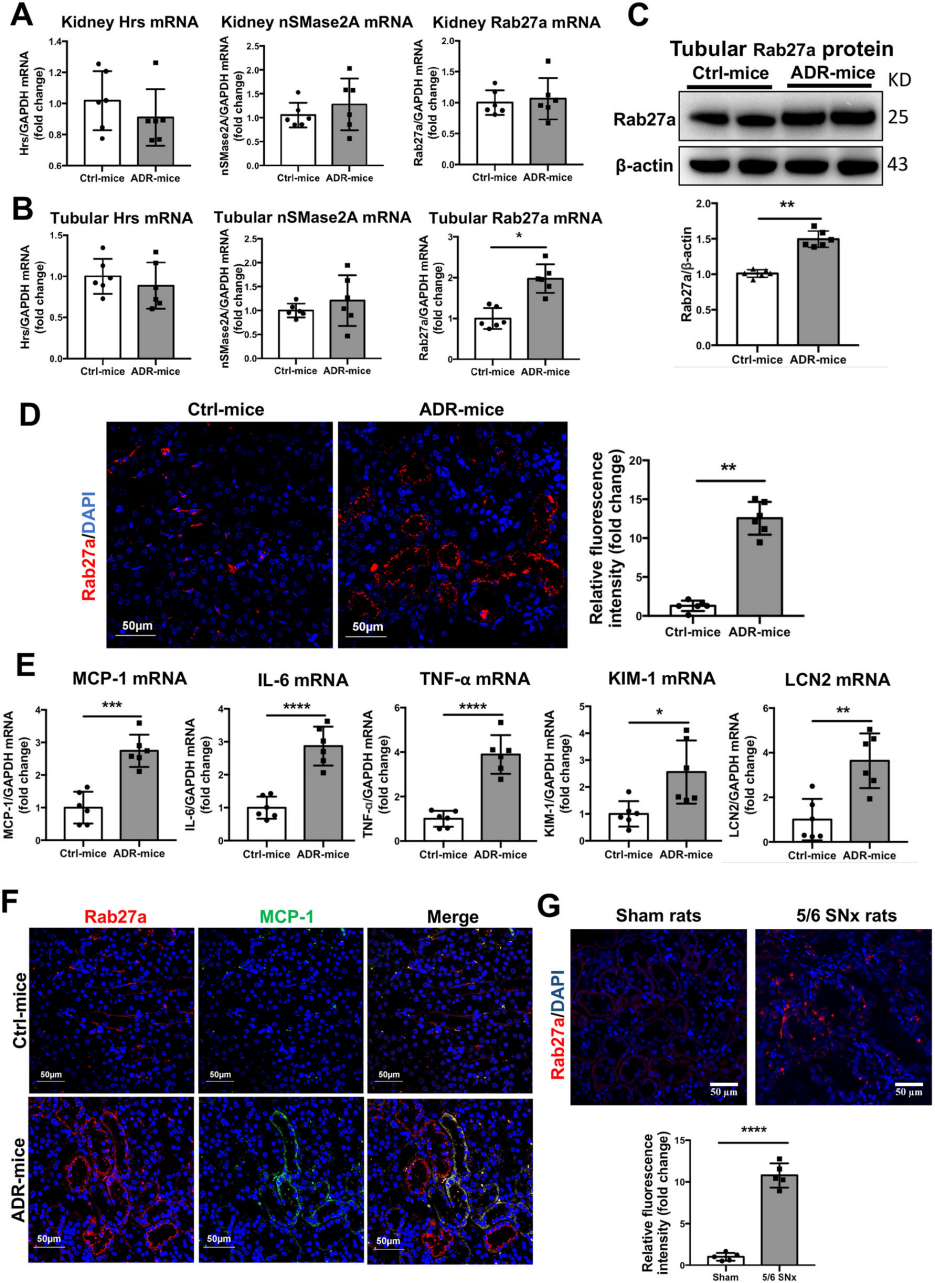
Tubular Rab27a as well as inflammatory response were induced during proteinuric kidney injury. **a** The expression of Hrs, nSMase2A, and Rab27a mRNA in renal cortex from mice with or without ADR injection. No significant difference was found in the mRNA expression between the two groups. The results are normalized to GAPDH and compared with Ctrl mice (represented by 1-fold). **b** Expression of Hrs, nSMase2A, and Rab27a mRNA in isolated tubules. Significant increase of Rab27a mRNA expression was shown in the tubular fractions of ADR-treated mice, normalized to GAPDH and compared with Ctrl mice (represented by 1-fold). **c** Representative western blotting and quantification of Rab27a protein in tubules from ADR-injected or control mice. The results are normalized to β-actin. **d** Representative images of immunofluorescence for Rab27a in kidney. Rab27a was expressed exclusively in TECs with significant upregulation in ADR mice compared to Ctrl. The bar graph shows fluorescence intensity from ADR-induced mice compared with levels in control mice (represented by 1-fold). Scale bar: 50 μm. **e** Expression of inflammatory cytokine (MCP-1, IL-6, and TNF- α) and tubular injury markers (KIM-1 and LCN2) in tubular fractions are normalized to GAPDH and compared with Ctrl mice (represented by 1-fold). **f** Representative immunofluorescent images of Rab27a (red) and MCP-1 (Green) in vivo. Scale bar: 50 μm. *p < 0.05, **p < 0.01, ***p < 0.001, ****p < 0.0001 vs Ctrl mice. Ctrl, control; ADR, adriamycin. *n* = 6 for each group of mice. **g** Representative images of immunofluorescence for Rab27a in kidney from 5/6 nephrectomy rats and sham rats. The bar graph shows fluorescence intensity from 5/6 nephrectomy rats compared with levels in sham rats (represented by 1-fold). Scale bar: 50 μm. ****p < 0.0001 vs sham rats. SNx, subtotal nephrectomy. *n* = 5 for each group of rats.

To explore potential molecules that involved in tubular exosome secretion during proteinuric kidney injury, we screened the key molecules candidates that are participated in exosome biogenesis and trafficking, including Hrs^25^, nSMase2A^26^, and Rab27a^27^. We found that mRNA expression of Rab27a, but not Hrs or nSMase2A, was highly expressed in tubular fractions isolated from ADR-injected mice compared with Ctrl mice (Fig. 2a, b). And western blot analysis confirmed the increased expression of Rab27a in isolated tubules (Fig. 2c). Next, subcellular distribution of Rab27a in the injured kidney was explored. Immunofluorescence staining showed that Rab27a expression was enhanced significantly which was mainly located at the apical membrane of tubular structures in ADR-injected mice, while very low expression of Rab27a at the basolateral side was observed in Ctrl mice (Fig. 2d). Meanwhile, enhanced inflammatory response and tubular injury were observed in tubules, as shown by RT-PCR detection of MCP-1 IL-6, TNF-α, KIM-1, and LCN2 in tubule fractions (Fig. 2e). Interestingly, immunofluorescence staining of the kidney showed the colocation or close location of those tubules expressing Rab27a and MCP-1(Fig. 2f). Moreover, higher expression of Rab27a was observed in tubules of 5/6 nephrectomy rats (Fig. 2g), which suggested that Rab27a is upregulated by albumin overload accompanied with tubules inflammation during proteinuric kidney injury.

### Albumin overload promoted exosome release and Rab27a expression in a dose-dependent manner

**Fig. 3:**
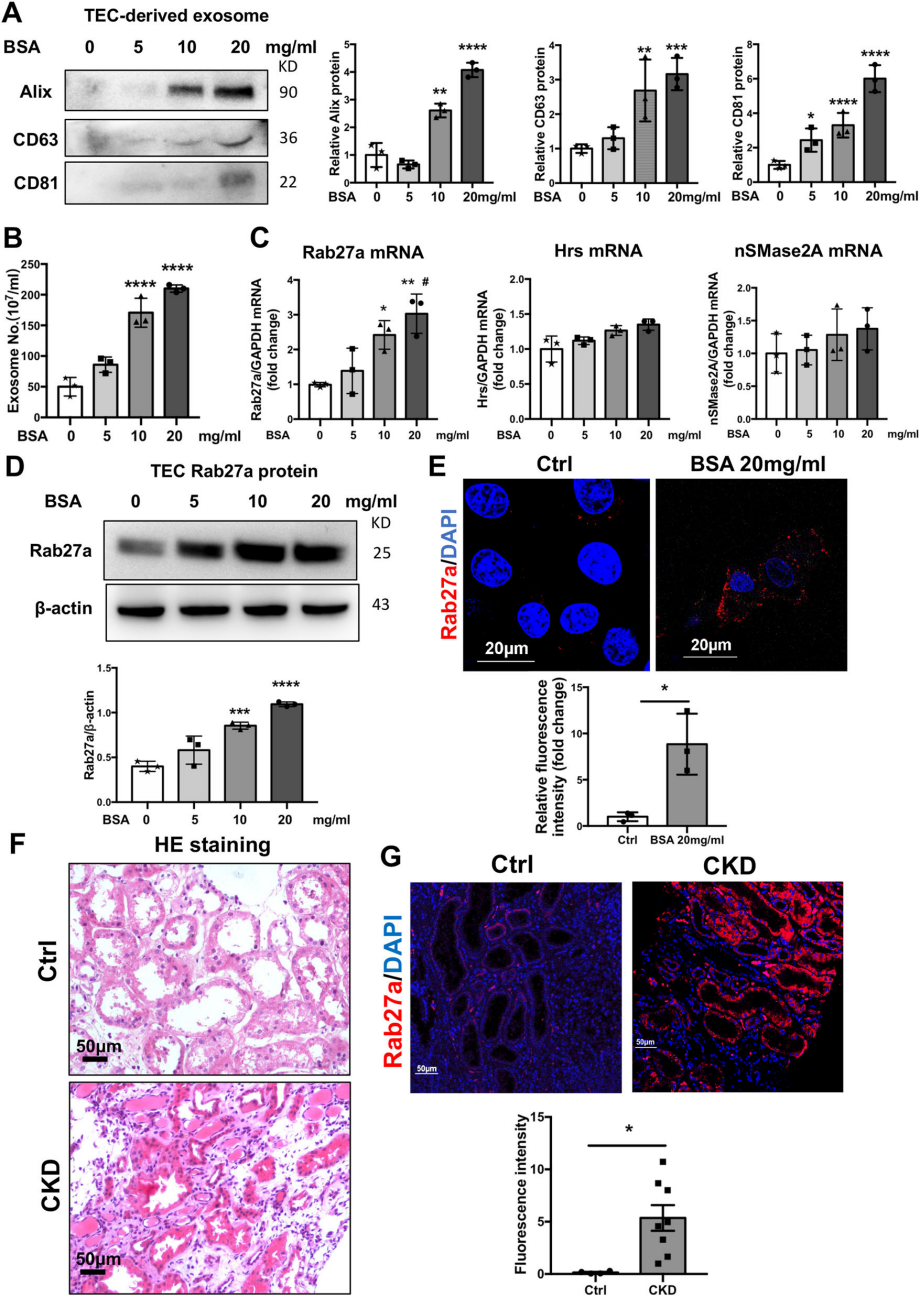
Albumin overload promoted exosomes secretion and Rab27a expression in a dose-dependent manner. **a**, **b** Quantification of purified exosomes released from TECs exosomes treated with different doses of BSA (0, 5, 10 and 20 mg/ml). Increased, secretion of exosomes was observed shown as representative western blotting of exosome markers (Alix, CD63, and CD81) (**a**) and quantification using EXOCET assay kit (**b**). **c** Expression of Hrs, nSMase2A, and Rab27a mRNA in TECs exposed to different doses of BSA detected by RT-PCR. The mRNA expression is compared to cells without BSA treatment (represented by 1-fold). **d**, **e** Rab27a protein in TECs with albumin treatment. Rab27a protein was upregulated by BSA treatment in a dose-dependent manner, as shown by western blotting and cellular immunofluorescence staining. The bar graph shows fluorescence intensity from 20 mg/ml-BSA-treated TECs compared with Ctrl group (represented by 1-fold). Scale bar: 20 μm. **f**, **g** HE staining and immunofluorescence staining of Rab27a expression in renal biopsies from controls (*n* = 4) (normal kidney tissue from patients of kidney cancer) and patients with CKD (*n* = 8). Severe tubular injury, protein casts (**f**) and higher expression of Rab27a in the tubules was observed in CKD patients compared with controls (**g**). Scale bars: 50 μm. Ctrl, control. *p < 0.05 compared to Ctrl. *p < 0.05, **p < 0.01, ***p < 0.001, ****p < 0.0001 vs TECs without BSA exposure. ^#^*p* < 0.05 vs TECs with 5 mg/ml BSA treatment. BSA, bovine serum albumin. Data presented as mean ± S.E.M. of three independent experiments.

To investigate the effect of albumin overload on exosome secretion, different doses of albumin was applied to cells in vitro. After 24 h of exposure for sufficient cellular uptake of albumin, TECs were cultured in medium without albumin for another 24 h allowing for the secretion of exosomes. Interestingly, albumin treatment increased exosome secretion in a dose-dependent manner as detected by EXOCET assay and western blot analysis of exosome markers, Alix, CD63, and CD81 in purified exosomes (Fig. 3a, b). In addition, both the mRNA and protein levels of Rab27a was increased with increasing amount of albumin applied to TECs, while no significant change was observed for Hrs and nSMase2. (Fig. 3c, d). Immunostaining also revealed the increased expression of Rab27a in TECs exposed to albumin compared to Ctrl (Fig. 3e).

Clinically, CKD patients with proteinuria (average 3.02 g/24 h, *n* = 8) were enrolled to detect the expression of Rab27a in kidney tissue. The clinical characteristics were list in Table 2. Histologically, severe tubular injury, and protein casts were observed in CKD patients by HE staining (Fig. 3f). Rab27a was detected with remarkable increased level in kidney tissue from CKD patients with proteinuria compared to normal kidney tissue from patients of renal cancer (*n* = 4) as the controls (Fig. 3g). Therefore, exosome secretion and Rab27a expression were enhanced significantly in TECs with increasing albumin exposure.

**Table 2:** Clinic characteristics of controls and chronic kidney disease (CKD) patients.

	Ctrl (*n* = 4)	CKD (*n* = 8)
Male	2 (50%)	4 (50%)
Age	32 (28–40)	54 (43.25–61)
BUN	4.8 (4.1–5.6)	9.3 (4.6–13.9)
SCr	70.5 (55.5–80)	142.5 (78.5–196.3)
UA	298 (258–336)	313 (269–367)
24 h proteinuria	0	4.82 (3.15–6.82)

Ctrl, normal kidney tissues from patients with renal carcinoma were used as controls; BUN, blood urea nitrogen; SCr, serum creatinine; UA, uric acid. CKD, chronic kidney disease, including patients with IgA nephropathy, membranous nephropathy, diabetic nephropathy and lupus nephritis.

### Albumin promoted exosome release via Rab27a and induced TECs injury as a paracrine or autocrine signal

**Fig. 4:**
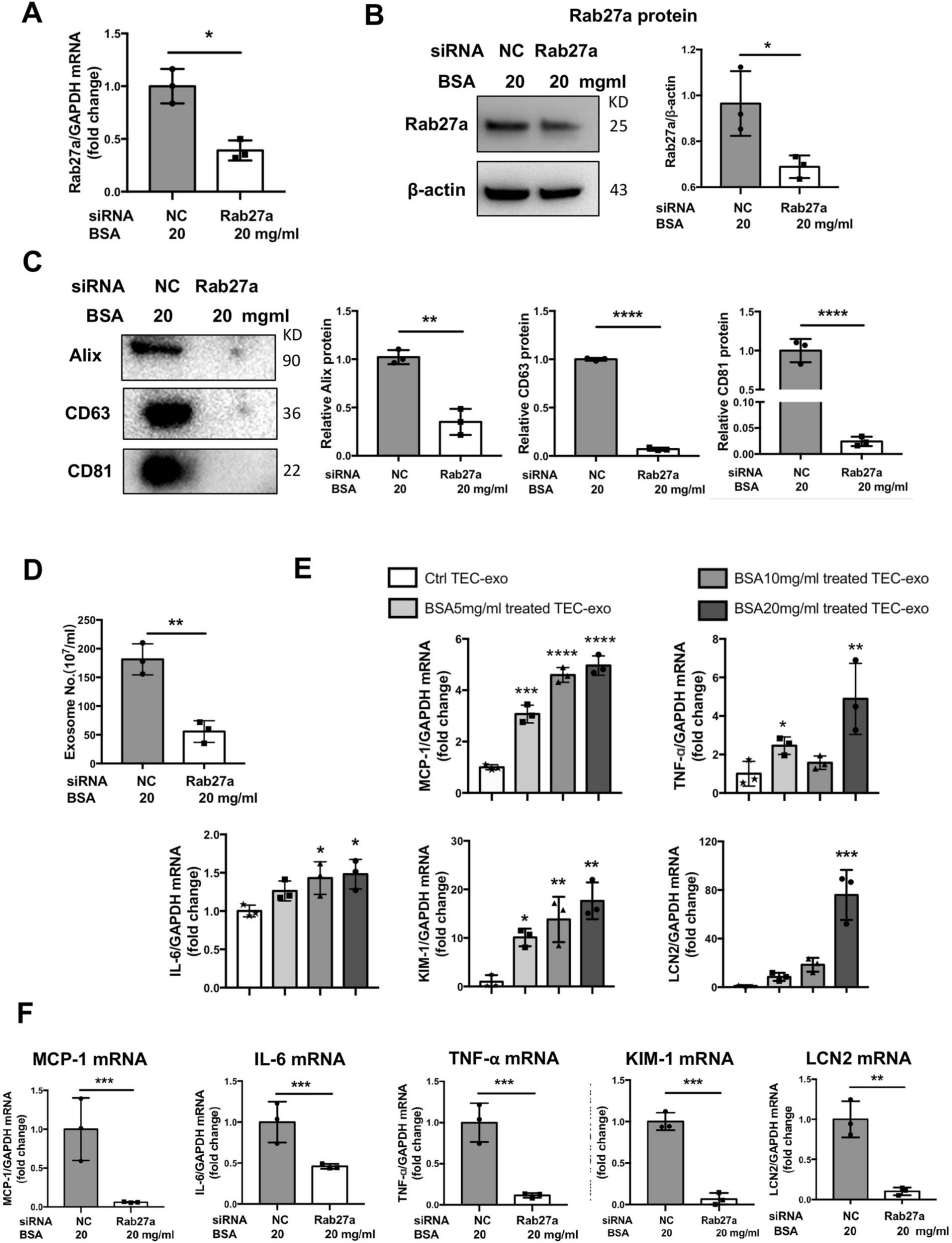
Albumin promoted exosomes secretion via Rab27a and induced TECs injury as a paracrine or autocrine signal. **a**, **b** Rab27a knockdown by siRNA transfection in the presence of BSA. Rab27a was efficiently knockdown at mRNA and protein level. *p < 0.05 vs TECs transfected with NC. **c**, **d** Quantification of exosomes isolated from cultured supernatant of TECs. Knockdown of Rab27a significantly reduced exosome secretion as detected by western blotting analysis of exosomal markers (Alix, CD63, and CD81) (**c**) and EXOCET assay (**d**). **p < 0.01, ****p < 0.0001 vs TECs transfected with NC. **e** Exosomes isolated from TECs exposed to different doses of BSA were applied to naïve TECs. The mRNA expression of inflammatory cytokine (MCP-1, TNF-α, and IL-6) and tubular injury markers (KIM-1 and LCN2) in exosome-treated TECs are normalized to GAPDH and compared with Ctrl-exosomes-treated TECs (represented by 1-fold). *p < 0.05, **p < 0.01, ***p < 0.001, ****p < 0.0001 vs TECs treated with Ctrl TEC-exo. **f** Expression of inflammatory cytokine and tubular injury marker in Rab27a knockdown-TECs in the presence of BSA are normalized to GAPDH and compared with NC-transfected TECs with BSA treatment (represented by 1-fold). **p < 0.01, ***p < 0.001 vs TECs transfected with NC. NC, negative control. Data presented as mean ± S.E.M. of three independent experiments.

To confirm the role of Rab27a in exosome secretion in TECs, Rab27a expression was silenced with siRNA transfection (Fig. 4a, b). Knockdown of Rab27a reduced the release of exosomes remarkably in TECs with albumin treatment (Fig. 4c, d), demonstrating that Rab27a is required for exosome secretion for albumin-treated TECs. Next, the effects of TEC-derived exosomes on both neighbored naïve TECs and the producing cells were explored. Exosomes secreted from TECs in the presence of BSA significantly elevated inflammatory cytokine (MCP-1, TNF-α, and IL-6) and tubular injury markers (KIM-1 and LCN2) mRNA expression in naïve TECs in a BSA dose-dependent manner (Fig. 4e). Interestingly, inhibition of exosomes secretion significantly repressed inflammatory response and tubular injury in the producing TECs (Fig. 4f). Overexpression of Rab27a with recombinant plasmid under albumin overload could slightly upregulate the inflammatory cytokines and tubular injury markers, but without reaching statistical significance indicating the capability of tubules in albumin handling through Rab27a-mediated exosomes could not be enhanced remarkably when it reached a certain level (Supplementary Fig. 1a, b). Thus, albumin overload promoted exosomes secretion via Rab27a, while secreted exosomes activated inflammation response in TECs as a paracrine or endocrine signal.

### Interferon regulatory factor 1 (IRF-1) is induced as the transcription factor of Rab27a in TECs

**Fig. 5:**
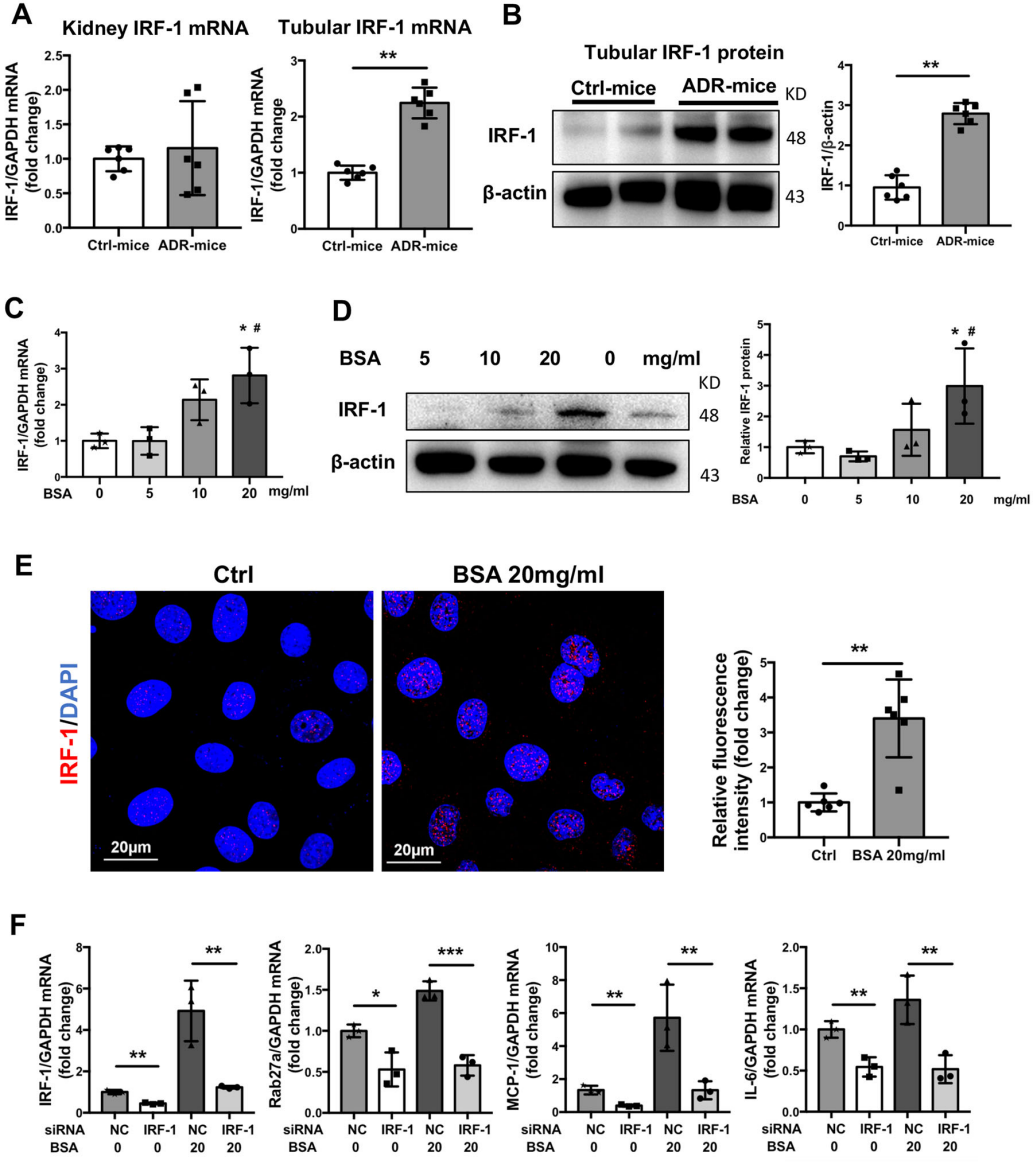
Interferon regulatory factor 1 (IRF-1) is induced as the transcription factor of Rab27a in TECs both in vivo and in vitro. **a**, **b** IRF-1 expression in ADR and Ctrl mice. IRF-1 mRNA expression was upregulated in isolated tubules, other than whole kidney in ADR mice compared to Ctrl (**a**). Western blotting analysis shown remarkable increased Rab27a protein in ADR mice (**b**) **p < 0.01 vs Ctrl-mice (represented by 1-fold). **c**–**e** IRF-1 expression in cultured TECs exposed to BSA. IRF-1 mRNA and protein were upregulated in TECs by BSA treatment in a dose-dependent manner. *p < 0.05 vs TECs without BSA exposure, ^#^*p* < 0.05 vs TECs treated with 5 mg/ml BSA (**c**, **d**). Immunofluorescence staining for IRF-1 (red) showed increased expression in both nuclei and cytoplasm (**e**). Scale bars: 20 μm. **f** Expression of IRF-1, Rab27a, and inflammatory cytokines in IRF-1 knockdown-TECs with or without albumin exposure. Ctrl-mice: control mice. **p < 0.01, ***p < 0.001 compared to NC (represented by 1-fold). Data presented as mean ± S.E.M. of three independent experiments. NC, negative control.

As previous studies reported, IRF-1 is one of the transcription factors that regulates Rab27a expression through binding directly to its promotor region^28^. To confirm the regulatory mechanism of Rab27a expression under proteinuric kidney injury, IRF-1 was analyzed in ADR-treated mice and BSA treated TECs. Interestingly, both mRNA and protein levels of IRF-1 were induced significantly in isolated tubules from ADR-injected mice despite the unremarkable change in the whole kidney (Fig. 5a, b). Consistent with the in vivo study, IRF-1 mRNA and protein were also induced in TECs stimulated with BSA in a dose-dependent manner (Fig. 5c, d). Similarly, immunostaining showed that albumin exposure increased nuclear location of IRF-1 (Fig. 5e). Silencing of IRF-1 with siRNA decreased Rab27a and inflammatory cytokines (MCP-1 and IL-6) expression in response to albumin exposure (Fig. 5f). Considering that KIBRA has been proven as an adaptor-like protein to prevent Rab27a from ubiquitination for degradation in kidney^29^, KIBRA siRNA was transfected in TECs to investigate the role of KIBRA in Rab27a expression. Knockdown of KIBRA attenuated upregulation of Rab27a protein in TECs exposed to albumin (Supplementary Fig. 2). These results suggested that IRF-1 was induced significantly as the transcription factor by albumin which consequently promoted Rab27a expression, and KIBRA might participated in stabilizing of Rab27a as well.

### Rab27a-dependent exosome secretion provided an alternative pathway to lysosome for albumin handling in TECs

**Fig. 6:**
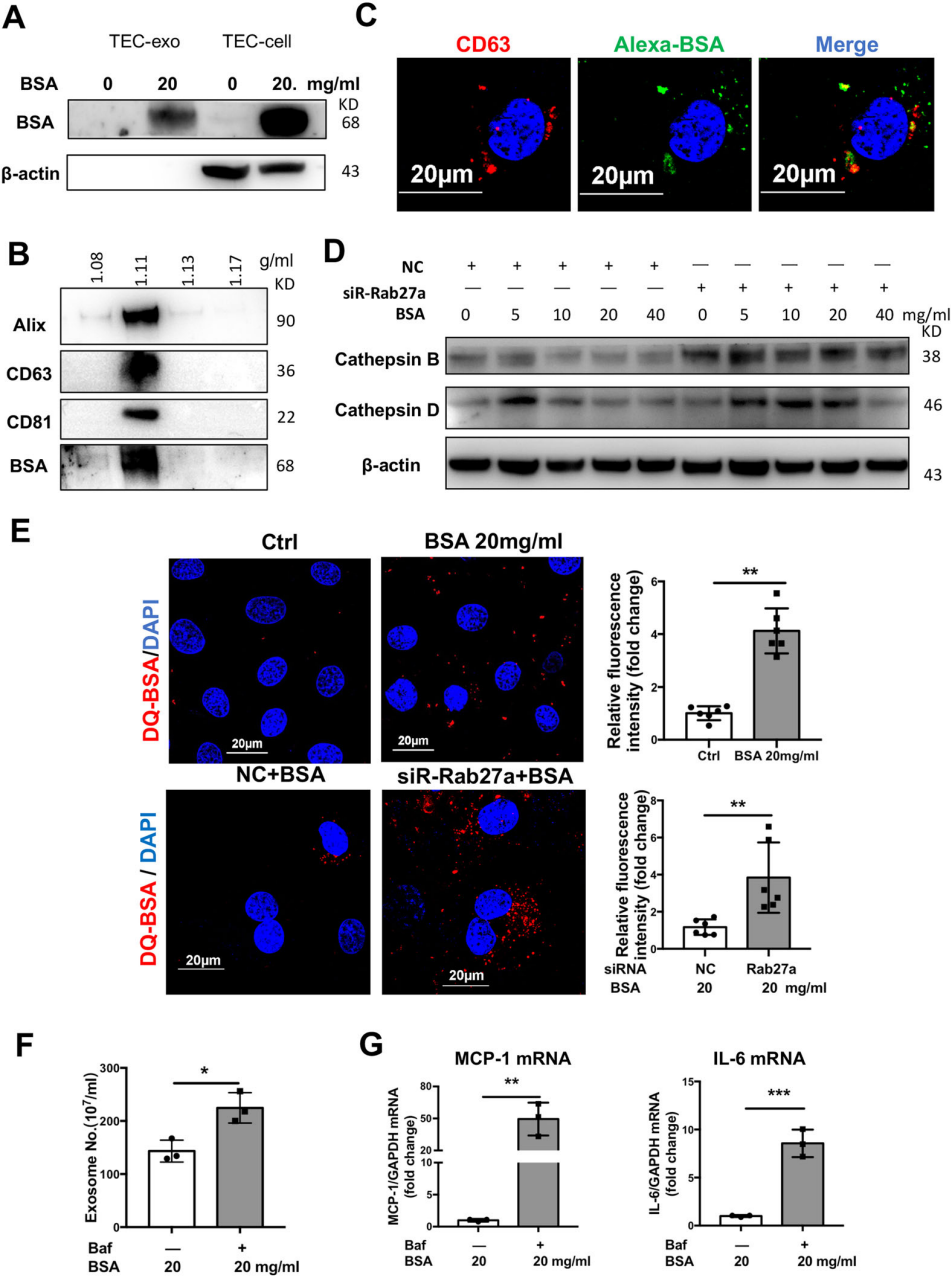
Rab27a-dependent exosome secretion provides an alternative pathway to lysosome for albumin handling in TECs. **a**, **b** Detection of BSA in purified exosome fraction from cultured TECs. After incubation with or without BSA for 24 h, the medium was replaced with albumin-free medium and cells were cultured for another 24 h allowing the secretion of exosomes. BSA was readily detected by western blot analysis in the cell and exosome fractions isolated by differential ultracentrifugation (**a**). BSA was detected in exosome fractions with density of 1.11 g/ml which was purified from OptiPrep density gradient centrifugation to exclude the possibility of protein contaminant (**b**). **c** Representative confocal co-localization of the MVB marker CD63 (red) and FITC-BSA (green). FITC-BSA was applied to TECs for 24 h and cells were fixed and immunostained with anti-CD63 antibody. Scale bars: 20 μm. **d** Western blotting of lysosome cathepsin B and D in cell lysates from TECs with Rab27a siRNA at different dose of BSA. **e** Representative confocal microscopy analysis of DQ-BSA (red) to show albumin degradation in TECs. DQ-Red BSA were added after BSA treatment for 20 h, cells were fixed and stained with DAPI (blue). Significant amount of degraded albumin (red) was detected in BSA overload TECs, while increased albumin degradation was observed with Rab27a siRNA group. Scale bars: 20 μm. **f**–**g** Exosome secretion and inflammation response in TECs with Lysosome inhibitor, Baf. Exosome producing (**f**) as well as inflammatory cytokine expression were enhanced by Baf treatment (**g**). Data presented as mean ± S.E.M. of three independent experiments.

To further explore whether exosome secretion was involved in albumin handling, exosomes were purified from albumin-free medium after a period of time for albumin uptake. BSA was readily detected in the exosome fractions as well as the producing cells (Fig. 6a). To confirm the findings, OptiPrep density gradient centrifugation was performed to purify exosome fraction to exclude protein contaminant. Immunoblot analysis revealed that exosome markers (Alix, CD63, and CD81) as well as BSA were predominantly detected in the fraction of exosomes with equivalent density of 1.11 g/ml, indicating that BSA could be loaded into exosomes and secreted to the extracellular space (Fig. 6b). Besides, co-distribution of CD63-positive MVBs and FITC-BSA further support the loading of albumin into MVB, suggesting that exogenous BSA might partly be processed through endosomes and released through the fusion of MVBs with plasma membranes (Fig. 6c).

Next, the relation of exosomes dependent albumin handling with lysosome degradation was explored. We observed slightly enhanced expression of lysosomal cathepsin B and D with low level of albumin exposure, while no continuing upregulation was observed with increasing amount of albumin applied, which may suggest lysosome insufficiency in conditions of albumin overload. Interestingly, lysosomal enzyme increased remarkably when exosomes secretion was inhibited by Rab27a siRNA (Fig. 6d). Besides, DQ-albumin, a dye-quenched fluorescent albumin was applied to visualize the degradation of albumin by the lysosome^23^. Albumin degradation was readily detected in BSA-treated TECs, while the proteolytic activity of lysosomes visualized by DQ-BSA fluorescence was significantly increased in Rab27a-knockdown TECs in the presence of BSA (Fig. 6e).

To assess whether inhibition of lysosome degradation could recover exosome secretion in albumin handling, lysosome inhibitor, Bafilomycin A1 (Baf) was applied to TECs. Interestingly, exosomes secretion was enhanced significantly as indicated by quantification of purified exosomes when lysosome degradation was inhibited (Fig. 6f). More importantly, increasing expression of inflammatory cytokines was found in BSA-exposed TECs with Baf pretreatment which was consistent with the accelerated cell injury caused by increased exosomes release (Fig. 6g).

These results indicated that exosomes were released and contribute to albumin excretion as an alternative response in TECs to lysosome degradation in condition of lysosome insufficiency.

### Inhibition of Rab27a dependent exosome secretion ameliorates proteinuric kidney injury

**Fig. 7:**
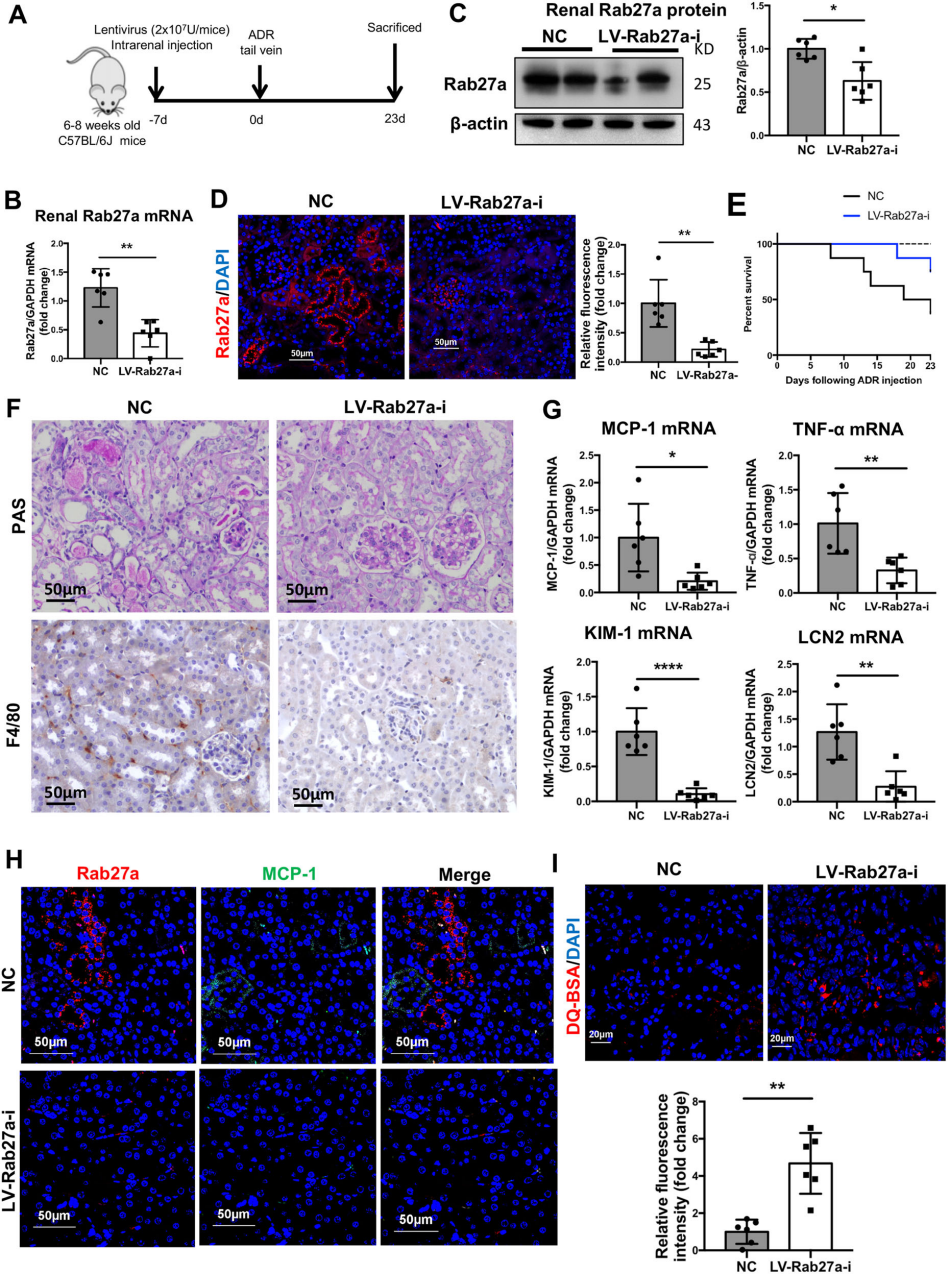
Inhibition of Rab27a dependent exosome secretion ameliorates proteinuric kidney injury. **a** A schematic review of in vivo experiment. Lentivirus Rab27a shRNA (Rab27a-i) or NC was administered to C57BL/6J mice through intrarenal injection. One week after lentivirus injection, mice received a single dose of ADR(18 mg/kg) administration through tail vein and were euthanized at 23 days. **b**–**d** Renal Rab27a expression in ADR mice with Rab27a shRNA treatment. Both mRNA and protein level of Rab27a were efficiently reduced by shRNA treatment. Expression of Rab27a in tubules were significantly decreased in renal sections from shRNA treated mice (red). Scale bars: 50 μm. **e** Survival curve showed ADR-induced mice received lentivirus Rab27a inhibitor had lower mortality compared with ADR-induced mice received NC. **f** Histologic changes (PAS staining) and F4/80 positive macrophage infiltration in the kidney. Rab27a knockdown remarkably alleviated tubule injury and interstitial inflammation. Scale bars: 50 μm. **g** The mRNA expression of inflammatory cytokines (MCP-1 and TNF-α) and tubular injury markers (KIM-1 and LCN2) from renal cortex of mice detected by RT-PCR are normalized to GAPDH and compared with NC mice (represented by 1-fold). **h** The expression of Rab27a and MCP-1 were evaluated using immunostaining. Images shown reduced Rab27a expression as well as MCP-1 expression in Rab27a knockdown mice. Scale bar: 50 μm. **i** Confocal microscopy analysis of degraded DQ-BSA (red) in renal cortex from ADR-mice with or without Rab27a knockdown. Scale bar: 20 μm. *p < 0.05, **p < 0.01, ****p < 0.0001 vs ADR-injected mice with NC administration. NC, negative control. *n* = 6 for each group of mice.

To explore the role of albumin handling by exosome secretion in renal inflammation during proteinuric kidney injury, C57BL/6J mice were administered with lentivirus Rab27a shRNA (Rab27a-i) or negative control intraparenchymal 1 week prior to ADR administration (Fig. 7a). As expected, lentivirus Rab27a shRNA pre-treatment inhibited mRNA and protein expression of Rab27a in kidney efficiently (Fig. 7b, c). Likewise, tubular Rab27a was significantly decreased as shown by renal immunofluorescence staining (Fig. 7d).

Impressively, knockdown of Rab27a expression significantly improved the survival of ADR-induced proteinuric mice (Fig. 7e). Rab27a knockdown ameliorated kidney injury as indicated by less tubular injury, protein casts and macrophage infiltration histologically (Fig. 7f). Besides, inflammatory cytokines MCP-1, TNF-α as well as tubular injury marker, KIM-1 and LCN2 expression were reduced remarkably in Rab27a knockdown group (Fig. 7g). Importantly, MCP-1 staining showed that inflammatory response was remarkably decreased in TECs in kidney of Rab27a knockdown mice (Fig. 7h).

To visualize the proteolytic capacity of the lysosomes in vivo, DQ-BSA was injected into mice via tail vein 1 h prior to being sacrificed. Impressively, increasing albumin degradation was found in ADR-injected mice with Rab27a knockdown (Fig. 7i). Taken together, these data suggested that inhibition of Rab27a-dependent exosome secretion ameliorated proteinuria kidney injury, probably through enhancing lysosome degradation of the toxic protein and repressing spread of exosomes containing albumin.

## Discussion

**Fig. 8:**
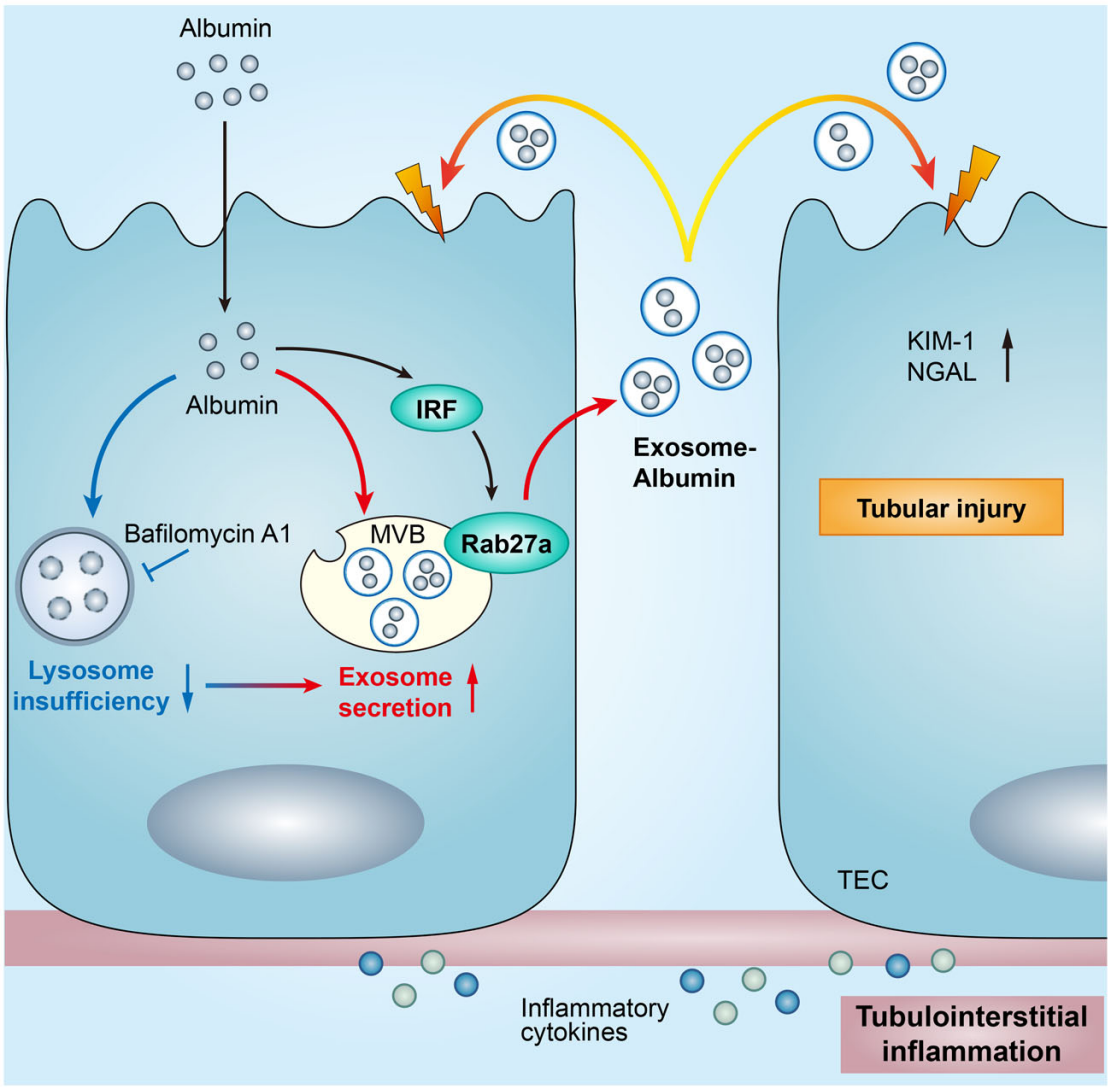
Working model. Our study revealed a novel approach of albumin handling via exosome secretion. Tubular epithelial cells (TECs) secreted exosomes carrying albumin via IRF-1/Rab27a, which constituted a coordinated approach to lysosome degradation. Inhibition of exosome release markedly enhanced albumin degradation, while lysosome inhibitor pretreatment promoted exosome secretion. Exosome secretion in response to albumin overload resulted in TECs injury and inflammation as the autocrine and endocrine signal during kidney injury.

Proteinuria is the most common manifestation of kidney disease, accumulating evidence showed that excessive albumin exposure elicited tubular cell injury and interstitial inflammation which determines the prognosis of renal disease^8,12^. In this study, we identified IRF-1/Rab27a dependent exosome release contributed to albumin handling in TECs as a novel alternative approach to lysosome degradation for albumin clearance. The secreted exosomes containing albumin may augment the proteinuria toxicity to TECs and accelerate tubulointerstitial injury (Fig. 8).

Firstly, we identified that exosome secretion increased significantly via IRF-1/Rab27a in TECs as the initial adaptive response to albumin overload, and KIBRA might participated in stabilizing of Rab27a as reported before^29^. Previous studies have suggested that stress conditions, such as hypoxia, proteinuria increased exosomes release from renal TECs^14,20^, while the underlying mechanisms remain unclear. In this study, we found that Rab27a expression increased exclusively in TECs with albumin exposure which was transcribed by IRF-1. Previously, Rab27a expression was detected in apical membrane of TECs which was involved in the regulation of polarized trafficking in epithelial cell^30,31^. Recently, Rab27 subfamily has been demonstrated to be involved in exosome secretion in cancer cells, dendritic cells by promoting the targeting of MVBs to the cell periphery and their docking at the plasma membrane^27^. In our study, we first described increasing Rab27a expression in diseased kidney which controlled exosome release by TECs. The finding suggested that Rab27a might be the interventional target to manipulate exosomes secretion in diseased kidney.

Secondly, we found that increased exosome secretion accelerated TECs injury as the paracrine and autocrine signals. Exosomes are increasingly being recognized as a novel form of cell-cell communication^1^. Studies from our group and others have observed the active secretion of exosomes in TECs under stress conditions^13–15^. However, the effect of the secreted TECs exosomes on the surrounding and parent cells deserve further investigation. We assume that TEC exosomes may travel along the route of the tubules and internalized by neighboring tubules which may change the status of the target cells. Interestingly, we found that exogenous exosomes indeed efficiently initiated inflammatory process of the receipt TECs, and inhibition of the endogenous exosomes alleviated the inflammation response and tubular injury. Similarly, exosomes induced cell proliferation, migration in both paracrine and autocrine fashion in breast cancer^32^. These facts support the idea that exosomes may take an active part in TECs injury as the paracrine and autocrine signals.

Thirdly, we revealed Rab27a-dependent exosome secretion as an alternative response to lysosome for overload of albumin in TECs. Under proteinuric condition, tubular handling and response to the excessive albumin has significant implications in kidney disease. Previous studies showed proteinuria stimulated tubular inflammation as well as proliferation responses especially when lysosome is insufficiency in processing albumin^33,34^. However, the underlying mechanism remained to be determined. Our study identified a novel response mediated by exosomes in albumin handling as demonstrated in proteinuric nephropathy model induced by ADR injection and 5/6 subtotal nephrectomy, as well as in CKD patients with proteinuria irrespective of the initial cause. Recently, emerging interest was raised regarding the capacity of exosomes as a means of alleviating intracellular stress conditions through secretion of harmful material in coordination with the autophagy-lysosomal pathway^16^. Impressively, we found that TECs eliminate excessive albumin via exosomes as demonstrated by the increasing loading of labeled albumin into MVB and its presence in the purified exosome fractions. Interestingly, inhibition of exosome release markedly enhanced lysosomal degradation both in vivo and in vitro, while lysosome inhibitor pretreatment increased exosome secretion when TECs are stressed with albumin. Hence, a balance response exists between lysosomes activity and exosome release in TECs for albumin handling. Similarly, Inhibition of LAMP1 and LAMP2 promotes exosome release in alcoholic liver disease^35^. A recent study in CKD GWAS and single-cell RNA sequencing revealed putative causal genes were enriched for proximal tubule expression and endo-lysosomal function^36^. Thus, those evidence points to a close relationship between lysosome and the biogenesis and secretion of exosomes which plays important role in kidney disease.

It has been established that albumin absorbed by megalin/cubilin/AMN could be sorted into lysosomes for degradation or be recycled from the primary urine by tubular transcytosis^10,37^. However, lysosomal degradation of reabsorbed proteins is a saturable process, inadequate degradation of reabsorbed proteins may contribute to tubular injury^38,39^. Our study revealed a novel response mechanism for tubular to clear albumin through exosome secretion which on the otherwise facilitated the spreading of toxic albumin to surrounding cells. It may explain the mechanism of tubular injury with increasing load of albumin and lysosome insufficiency. The initial response to excrete excessive albumin by TECs lead to a maladaptive outcome because of the reuptake of exosome-albumin. However, how the fate of albumin toward lysosome degradation or secretion in exosomes is regulated still need further investigation.

Finally, we identified that manipulating the approach of albumin handling in TECs may represent a novel approach of ameliorating proteinuric kidney injury. Proteinuria correlates with the degree of tubulointerstitial injury and predict the likelihood of progression for human kidney disease. Thus, reduction of proteinuria is considered to be the major therapeutic target for slowing nephropathy progression^40^. Interestingly, we found that in the same level of proteinuria in ADR-mice and in vitro cell culture, repression of exosome release significantly attenuated tubular cell injury and inflammation. Thus, manipulate the response of TECs to excessive albumin to reduce the toxicity of protein might represent a novel direction for therapy of kidney disease in addition to lowering albuminuria.

In conclusion, we have demonstrated that IRF-1/Rab27a mediated exosomes secretion in TECs with albumin overload promoted tubular injury and tubulointerstitial inflammation as the paracrine and autocrine signals. Rab27a-dependent exosome secretion represents a coordinative response to lysosome degradation for albumin clearance. Repression of exosome secretion may become a logical therapeutic strategy to prevent disease progression in proteinuric nephropathies.

## Supplementary information

Supplementary Figure legends

Supplementary Figure 1

Supplementary Figure 2
