# Supplementary material for: Rab27a dependent exosome releasing participated in albumin handling as a coordinated approach to lysosome in kidney disease
Source: Cell Death Dis. 2020 Jul 8;11(7):513. doi: 10.1038/s41419-020-2709-4 (PMC7343869; doi:10.1038/s41419-020-2709-4)
Supplement: Supplementary file 1 — Supplementary Figure legends [file 41419_2020_2709_MOESM1_ESM.docx]

**Supplemental Figure 1.** Rab27a overexpression in TECs with albumin exposure. A. Rab27a was overexpressed by plasmid transfection in the presence of BSA. B. Expression of inflammatory cytokine and tubular injury marker in Rab27a overexpressed-TECs in the presence of BSA are normalized to GAPDH and compared with NC-transfected TECs (represented by 1-fold). ***p<0.001 vs TECs transfected with NC. NC, control. Data presented as mean ± S.E.M. of three independent experiments.

**Supplemental Figure 2.** Knockdown KIBRA attenuated Rab27a protein level in TECs exposed to albumin. *p<0.05 vs TECs transfected with NC. NC, negative control.
