# Supplementary figures and images for: Rab27a dependent exosome releasing participated in albumin handling as a coordinated approach to lysosome in kidney disease
Source: Cell Death Dis. 2020 Jul 8;11(7):513. doi: 10.1038/s41419-020-2709-4 (PMC7343869; doi:10.1038/s41419-020-2709-4)

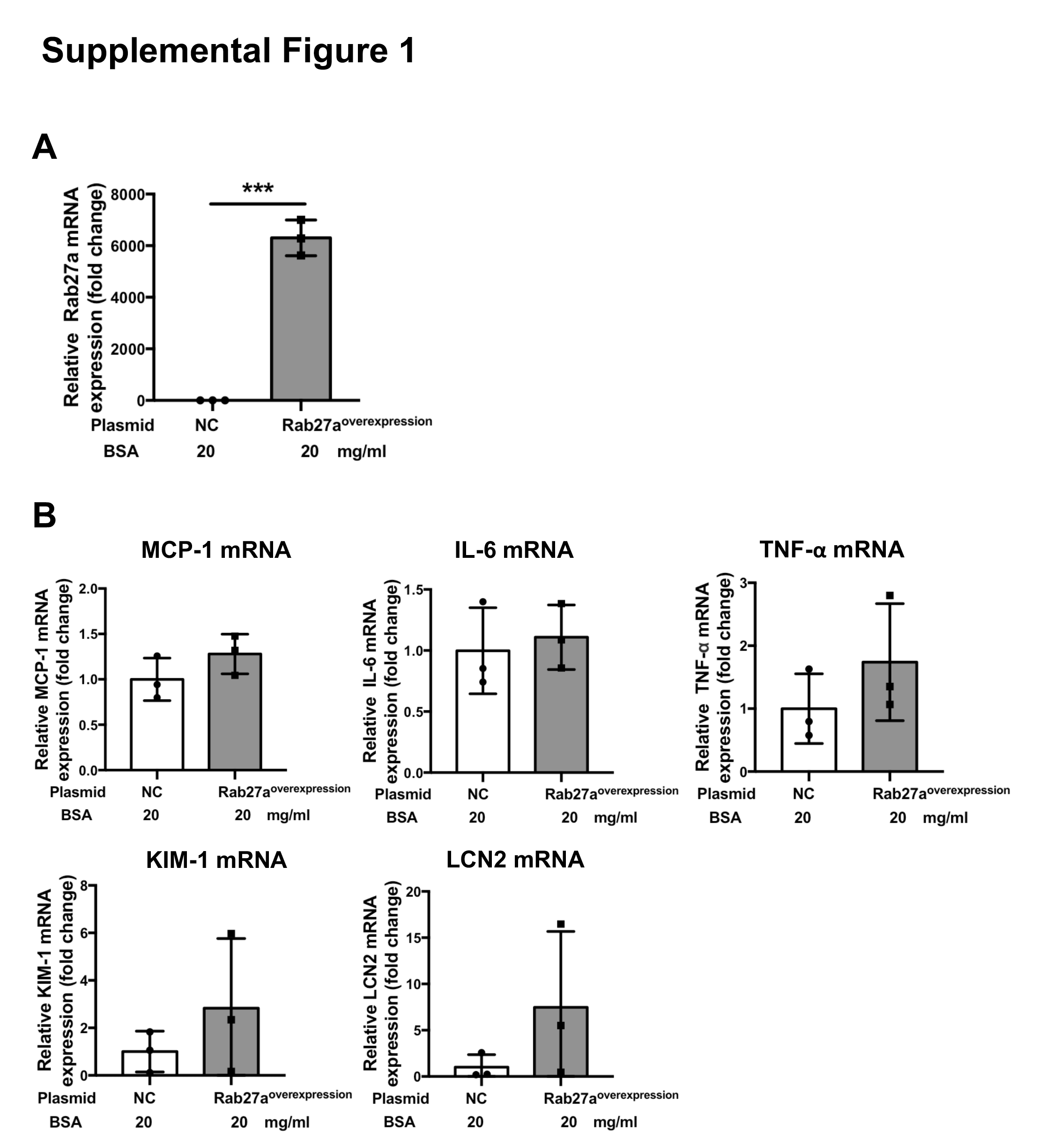

Supplement: Supplementary file 2 — Supplementary Figure 1 [file 41419_2020_2709_MOESM2_ESM.tif]

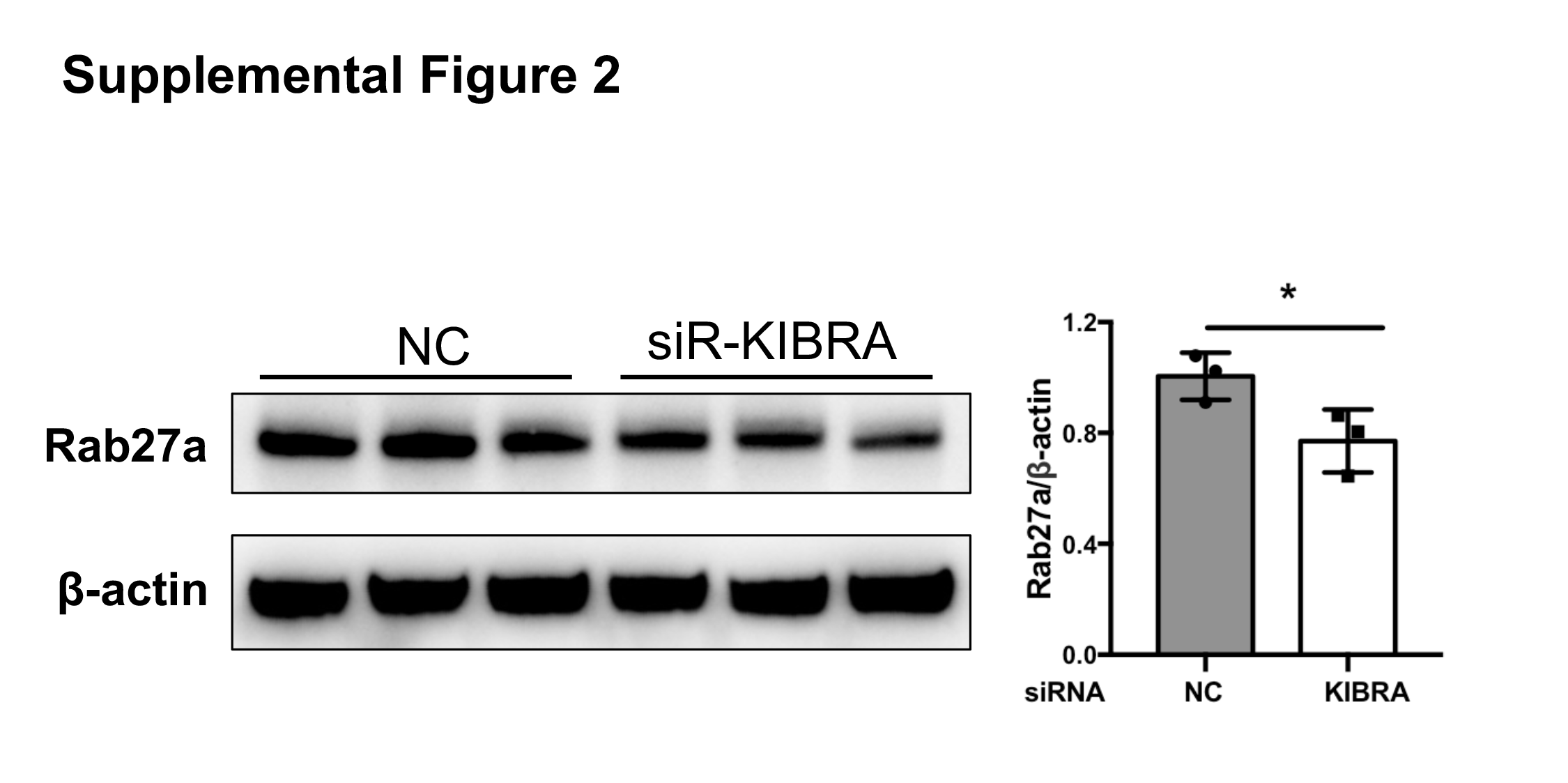

Supplement: Supplementary file 3 — Supplementary Figure 2 [file 41419_2020_2709_MOESM3_ESM.tif]
